# Purification and antigenic detection of O-specific polysaccharides of *Salmonella enterica* serovar Paratyphi A isolate from Pakistan: an emerging threat

**DOI:** 10.1186/s40064-016-3643-x

**Published:** 2016-11-10

**Authors:** Zainab Rahmat, Aamir Ali, Yasra Sarwar, Muhammad Salman, Abdul Haque

**Affiliations:** 1 National Institute for Biotechnology and Genetic Engineering (NIBGE), Faisalabad, Pakistan; 2 Department of Biotechnology, Abdul Wali Khan University, Mardan, Pakistan; 3 Department of Pathology, The University of Faisalabad, Sargodha Road, Faisalabad, Pakistan

**Keywords:** Antigenicity, Conjugate vaccines, Lipopolysacchrides, OSP, *Salmonella* Paratyphi A

## Abstract

**Background:**

Paratyphoid fever caused by *Salmonella enterica* serovar Paratyphi A is becoming a serious health problem in Asian countries particularly Pakistan, China and India and situation is aggravated by current unavailability of a licensed vaccine. This study was designed to purify the O-specific polysaccharides (OSP) produced by an isolate of *Salmonella* Paratyphi A from Pakistan and detect antigenicity of extracted lipopolysaccharide (LPS) and purified OSP pioneerly in South Asian region as candidate for conjugate vaccine preparation.

**Results:**

*S*. Paratyphi A isolates were identified through PCR using primers of *fliC*-*a* gene (329 bp) and confirmed via nested PCR using *fliC*-nested primers (289 bp). Yield of the LPS of *S*. Paratyphi A isolate was 40 mg/L of the bacterial culture using hot phenol method. The purified LPS revealed the characteristic ladder like pattern of *S*. Paratyphi A LPS on SDS-PAGE with silver staining. Purified OSP obtained by acid hydrolysis yielded 23 mg/L of culture broth and was not detected by silver staining. Antigenic interaction of the purified LPS and OSP with hyper immune mice sera was confirmed by single precipitin line evaluated through immunodiffusion assay. The antigenicity was found well intact.

**Conclusions:**

The purified antigenic OSP from *S.* Paratyphi A may have the potential to be coupled with a carrier protein to develop low cost conjugate vaccine candidates against *S.* Paratyphi A in paratyphoid endemic regions.

## Background

Paratyphoid fever is an emerging human invasive ailment of endemic regions of Southeast Asian developing world, caused by *S.* Paratyphi A and C. Incidence rate of Paratyphoid is about 150 cases/100,000 persons/year in Pakistan, India and China. Infants, school aged children in resource poor areas and travelers experience the greatest burden of the disease in Asia (Crump and Mintz 2010). Early and reliable detection is prerequisite to deliver patient care, therefore molecular detection methods have been developed for discrimination of this pathogen from other closely related enteric pathogens (Hirose et al. 2002; Ali et al. 2008; Kumar et al. 2010). Since, *S*. Paratyphi A is a human-restricted pathogen, the targeted vaccination of those groups at high risk may reduce paratyphoid disease burden substantially (Sahastrabuddhe et al. 2013).

The licensed vaccines are available against typhoid like Vi polysaccharide (ViPS) and Ty21a, however both of them have limited efficacy and they offer minimal cross-protection against paratyphoid infection sand cannot be given to children less than 2 years of age. Thus current unavailability of licensed vaccine against *S*. Paratyphi A focused the development of vaccine based on antigen of this bacterium (Konadu et al. 2000; Bhutta et al. 2014).

The LPS, surface polysaccharides of gram-negative bacteria can serve both virulence factor as well protective antigens. The O-specific polysaccharide is immuno-dominant part of LPS molecule and is obtained by acid hydrolysis of these LPS. Although OSP enhance immune system poorly being T cell independent antigens, however when combined with carrier protein these are capable of providing long lasting immunity (Arndt et al. 2014). Thus, OSP of *S*. Paratyphi A is best candidate for vaccine development. In a previous report *S*. Paratyphi A isolated from China was conjugated with a carrier protein (Ali et al.^2014^).

This study is based on isolation of *S.* Paratyphi A isolates from endemic area of South-Asian subcontinent, its molecular identification by species specific PCR followed by extraction of LPS and purification of OSP. The ultimate goal was to detect the in vitro antigenicity of purified LPS and OSP as candidate for vaccines. From the South-Asian region, this is first report to evaluate the antigenic potential of OSP of *S*. Paratyphi A as a vaccine candidate which can be effectively used with a suitable carrier protein as conjugate vaccine.

## Methods

### Screening and production of *S.* Paratyphi A cell mass

An isolate of *S.* Paratyphi A (SPA-1) was taken from National Institute for Biotechnology and Genetic Engineering (NIBGE, Faisalabad, Pakistan stock culture), previously isolated from local hospitalized patients. The *S.* Paratyphi A was grown on tryptic soy broth (TSB) (Merck, Cat. # 100,800) at 37 °C and then subcultured on MacConkey (Merck, Cat.# 105465) agar plate with overnight incubation at 37 °C. DNA extraction of overnight grown culture in TSB was carried out by chloroform-isoamyl alcohol method. Regular PCR was performed using a 25μL reaction mixture containing 5 µl of DNA template, 5 U *Taq* polymerase, 1.5 mM MgCl_2_, 50 nM dNTPs, and 40 pM of each primer. In nested PCR, 5 µl of regular PCR product was used as template (Hirose et al. 2002; Ali et al. 2008). The two set of primers used are given in Table 1. For regular PCR, initial denaturation was at 94 °C for 4 min followed by 30cycles of: denaturation at 94 °C for 1 min, annealing at 48 °C for 1 min and extension for 1 min. Conditions for nested PCR were similar except for annealing temperature which was 55 °C. The amplification products were electrophoresed on 2% agarose gel, stained and photographed by UV transilluminator Eagle Eye (Strata gene).

**Table 1:** Primers used in regular and Nested PCR detection of *S.* Paratyphi A

PCR	Primers	Gene	Sequence (5′–3′)	Amplicon size (bp)
Regular	fliC-s fliCa-as	*fliC*-a	AATCAACAACAACCTGCAGCG TAGTGCTTAATGTAGCCGAAGG	329
Nested	N-fliC-s N-fliCa-as	*fliC*-a	GCAGCGTGTGCGTGAACTGGCGG GACTTCGCTCTTCACATCATAT	289

The confirmed *S*. Paratyphi A isolate (SPA-1) was inoculated (6%, v/v) to a 20 L fermentor (Biostat C) containing TSB medium. The fermentation was controlled at a temperature of 32 °C with 30% dissolved oxygen and pH 7 for 11 h. The growth was supplemented with 25% sterilized glucose solution after 7 h, to enhance the carbohydrate yield. The bacterial cells were killed by addition of 1% formalin and harvested by centrifugation (7000*g*, 4 °C, 40 min).

### Purification of lipopolysacchrides (LPS) of *S.* Paratyphi A

Harvested cell pellet was processed for LPS extraction by hot-phenol method (Westphal and Jann 1965) with minor modifications. The cell pellet was suspended in 1 L distilled water with slow stirring at 4 °C overnight. Phenol (90%) was added (equal volume) and equilibrated at 68 °C. After vigorously stirring at 68 °C for 30 min, the mixture was cooled on ice bath for 30-45 min with stirring till temperature dropped to approx. 10 °C. The upper aqueous layer was collected by centrifugation (6000×*g*, 50 min, 10 °C). Extraction was repeated after addition of de-ionized water and the mixture was centrifuged again (6000×*g*, 50 min, 10 °C). The water layer was separated and combined with previous water layer. For nucleic acids precipitation, 0.01 M sodium acetate, 0.002 M calcium chloride and 25% ethanol were added to water layer and stirred at 4 °C overnight. The supernatant was collected by centrifugation (9200×*g*, 1 h, 10 °C), brought to 75% ethanol using pure ethanol, and stored at 4 °C overnight. The LPS was precipitated by centrifugation (6500×*g*, 4 °C, 1 h), dissolved in pyrogen free water and dialyzed (cut off 6000–8000 daltons) against pyrogen free water and lyophilized as crude LPS. The purity of crude and purified LPS was determined by measuring the level of possible contaminants: Nucleic acid contamination was measured spectrophotometrically (LPS in 1 mg/ml in 1% SDS solution) using formula A_260_/20 × 100, whereas, the level of protein contamination was determined by Bradford assay. The purified LPS were collected by dissolving in buffer (200 ml of 2 mM-MgSO_4_, 50 mMTris-Cl, pH 7.6), dialyzing after the addition of DNase (Promega Art. # M198 A), RNase (Roth Art. # 71561) and proteinase K (Roth Art. # 75281) followed by ultra-centrifugation (96,000×*g*, 4 h, 4 °C) as described earlier (Ali et al. 2012; Salman et al. 2015).

### OSP purification through Acid hydrolysis

Acid hydrolysis of the purified LPS was done to purify O-antigen chain as previously described (Chu et al. 1991). Briefly, the LPS were treated with 1% acetic acid as 10 mg/ml and incubated in boiling water for 90 min. The pH was brought up to 7.0 with 1 N sodium hydroxide. Ultracentrifugation (64,000*g* for 5 h at 10 °C)of the mixture was done after addition of one drop of 1 M calcium chloride. The supernatant containing the OSP was dialyzed against pyrogen free water and freeze-dried. The purified LPS and OSP were electrophoresed on SDS-PAGE (4% stacking gel, 12% resolving gel) and visualized with silver staining (Ali et al. 2012).

### Antigenic evaluation

Hyper immune sera were raised by injecting mice with formalin killed whole cell *S.* Paratyphi A for 3 weeks with 3 doses/week (Ali et al. 2012). Blood was collected by heart puncture after a week of the last dose and the serum was isolated. The study was approved by Institutional Ethical Committee and all applicable guidelines for the care and use of animals were followed. The antigenic detection of the purified polysaccharides was done by immunodiffusion assay in vitro on 1% agarose (Ouchterlony et al. 1986). Different dilutions (1/2, 1/4 and 1/8) of the raised hyper immune sera were checked for precipitation against different concentrations of LPS/OSP (1000, 500 and 250 μg/ml in saline). The slides were stained with Coomassie blue and visualized the result by unaided eye.

## Results

### Screening and bacterial cell mass production

**Fig. 1:**
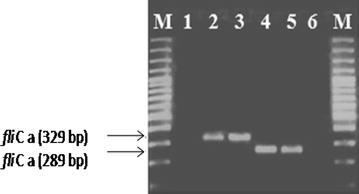
Molecular detection of *S*. Paratyphi A *Lane 1* and *8* Molecular weight marker, *Lane 2* and *7* Negative control, *Lane 3* and *4* Regular PCR of *S.* Paratyphi A *fliC*-*a* gene with product size of 329 bp, *Lane 5* and *6* Nested PCR of *S.* Paratyphi A *fliC*-*a* product size of 289 bp

Transparent, smooth, round and moist colonies, typical of *Salmonellae* were observed on MacConkey agar plate. Amplification of 329 bp fragment by regular PCR (Fig. 1 lanes 3, 4)and of 289 bp fragment by exploiting the internal primers of *fliC*-*a* gene in nested PCR (Fig. 1 lanes 5–6) confirmed the identification of *S.* Paratyphi A isolate. There was no amplification in negative control (without template DNA). These results demonstrated the sensitive detection of our *S.* Paratyphi A isolate from other related enteric pathogens as previously reported (Ali et al. 2008).

**Fig. 2:**
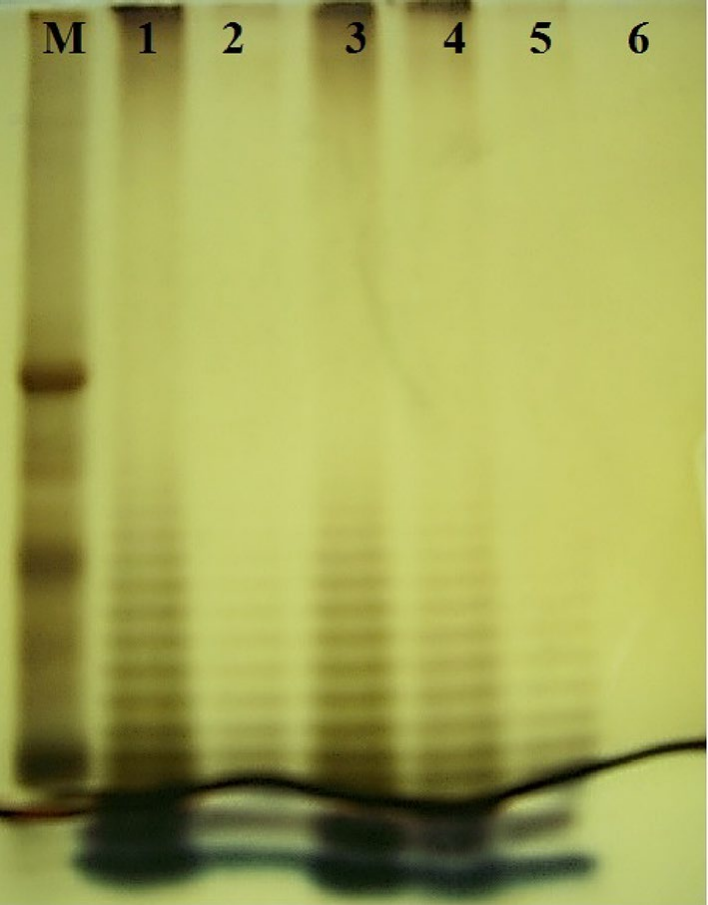
SDS-PAGE for LPS and OSP detection Lane M Molecular Weight marker, *Lane 1–5* contains different amounts of *S*. Paratyphi A LPS in 5, 1, 5, 2.5 and 1 µg respectively shown in ladder like pattern, *Lane 6* contains 10 µg of *S*. Paratyphi A OSP not visible by silver staining

*S*. Paratyphi A fermentation (20 L) yielded 187 g of wet cell pellet. The yield of pure LPS was 40 mg/L of the culture. This yield is comparatively less than reported yield of 150 mg/L by Micoli et al. (2013) who used a different downstream purification method at industrial scale in 30 L fermentor whereas yield is higher than 3.3 mg/L mentioned in another report (Kothari et al. 2014). The difference in yield of OSP was possibly due to multiple factors including polysaccharide content of each bacteria grown in different laboratories under different experimental settings. The purified LPS were electrophoresed on SDS-PAGE (Fig. 2 lanes 2–5) and showed characteristic ladder like pattern of *S.* Paratyphi A LPS as previously reported (Ali et al. 2014). Thus procedure adopted here can be scaled up to yield important enteric antigens for subsequent use in vaccine preparation.

### Paratyphi A OSP preparation

The freeze-dried OSP weighed as 23 mg/L of the culture not detected by silver staining on SDS-PAGE (Fig. 2 lane 6). It indicated that complete removal of lipid A had been achieved by ultracentrifugation. These findings are in accordance with already published data (Ali et al. 2012). The purified LPS and derived OSP of *S.* Paratyphi A were found to contain only 0.5% DNA and 1% protein contaminations (Table 2), well within permissible limits by World Health Organization (Tsai and Frasch 1982), strengthening the use of these OSP for vaccine production.

**Table 2:** Quality control assay for contamination of DNA and proteins in *S.* Paratyphi A LPS and OSP

Sample	Nucleic acid concentration (%)	Protein concentration (%)
*S.* Paratyphi A crude LPS	12	9.5
*S.* Paratyphi A purified LPS	2.5	1.9
*S.* Paratyphi A OSP	0.5	1

### Antigenic detection

**Fig. 3:**
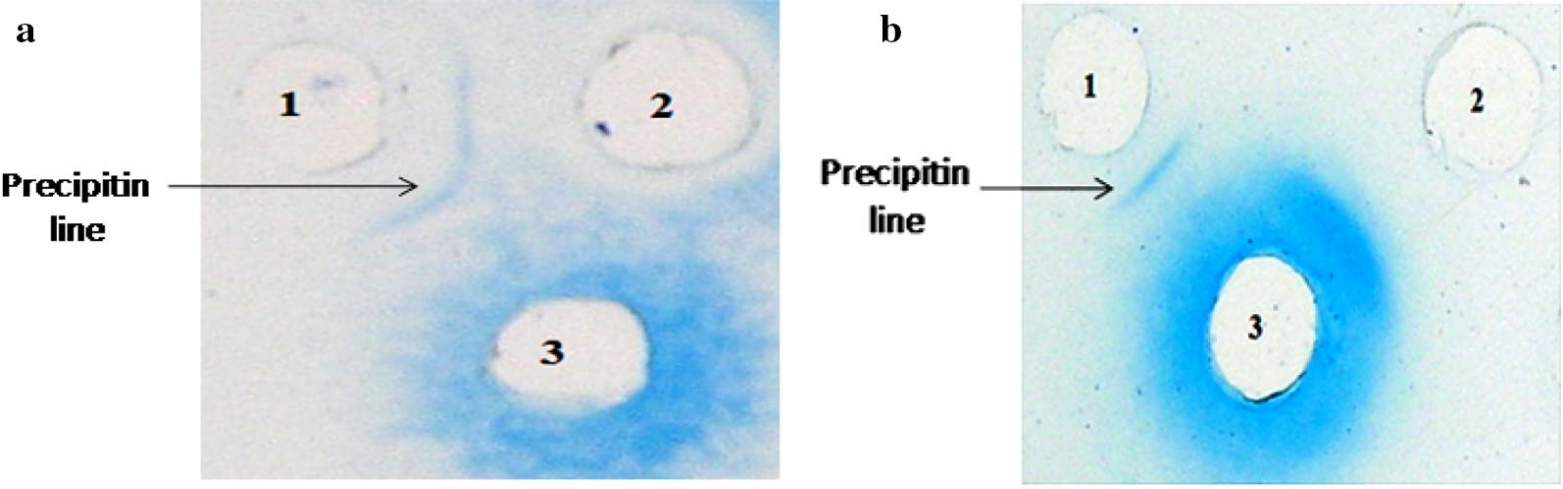
**a** The antigen antibody interaction in vitro, LPS of *S*. Paratyphi A by immunodiffusion assay. Well 1 LPS (250 μg/ml) in normal saline, well 2 and 3 shows Mice serum (1/8 time diluted polyclonal antibodies). The *arrow* shows precipitin line between *S*. Paratyphi A LPS and hyper immune mice serum raised against *S*. Paratyphi A, indicating LPS are antigenically active. **b** Antigenic evaluation of OSP against hyper immune mice sera by immunodiffusion assay Well 1 OSP (250 μg/ml) in normal saline, well 2 Negative control (normal saline), Well 3 Mice serum (1/8 time diluted polyclonal antibodies). The *arrow* shows precipitin line between *S*. Paratyphi A OSP and hyper immune mice serum raised against *S*. Paratyphi A, indicating antigenicity well maintained during purification procedure

The antigenicity of the purified LPS was detected in vitro and a clear precipitin line was formed between antigens (LPS/OSP) and antibodies (Fig. 3a, b, explicit by arrows) which assured that LPS/OSP of *S.* Paratyphi A were antigenically active. These results strongly suggested that OSP derived from these LPS have potential to be used in preparation of conjugate vaccines against *S*. Paratyphi A.

## Discussion

The increasing frequency of *S*. Paratyphi A in Asian continent highlights the need for a bivalent vaccine that protects against both *S*. Typhi and *S*. Paratyphi A. Vi capsular polysaccharide vaccines and a live attenuated (Ty21a) vaccine are licensed to provide protection against typhoid infection but licensed vaccines against *S*. Paratyphi A infection are scanty (Konadu et al. 1996). The Vi vaccine is licensed for use in children two years old and above; new generation conjugate vaccines are being developed and target use in infants less than two years old as well as older children (Ouchterlony et al. 1986). Several groups including the International Vaccine Institute (Seoul, Republic of Korea) are developing bivalent enteric fever vaccines which combine Vi conjugate (*S.* Typhi component) with O-specific polysaccharide conjugate (OSP) of *S.* Paratyphi A.

The lipid A part of the LPS is toxic so it is necessary to remove it from the OSP before using the OSP as a vaccine antigen. The rhamnose in trisaccharide backbone (rhamnose, mannose, and galactose) of *S*. Paratyphi A serovar specific OSP is partially O-acetylated at C-3 and this O acetylation is essential for inducing an antibody response to the OSP (Arndt et al. 2014). It is therefore important that the purification process retains the O-acetyl groups. These antigens alone are poorly antigenic and do not elicit proper immune response as only humoral immunity is evoked.

In case of *S.* Paratyphi A, unlike *S*. Typhi, there is no capsular covering so the only antigen available is OSP. The OSP has been exploited as target for conjugate vaccines against many diseases because of their immunogenicity. The OSP of *S*. Typhi derived from its LPS by acid hydrolysis have been conjugated to tetanus toxoid and bovine serum albumin (BSA). These conjugates were found to be immunogenic against typhoid fever (WHO: 1994). By conjugating OSP of *S*. Typhi with carrier protein like diphtheria toxoid (DT) has resulted in superior antibody responses, thus becoming a potential candidate in vaccine preparation (Ali et al. 2012). There are some other reports of OSP based conjugate vaccines. For example polysaccharides O:9, 12 specifically purified from *S*. Typhi was conjugated with tetanus toxoid and BSA (Aron et al. 1993), *Escherichia coli* O157 OSP was conjugated with a number of carrier proteins including exotoxin C of *Clostridium welchii*, BSA, rEPA (Konadu et al. 1994) and Shiga toxin 1 B subunit (Konadu et al. 1999) while the OSP of *Shigella dysenteriae* type 1 was linked with tetanus toxoid (Chu et al. 1991).

The OSP of *S*. Paratyphi A is a virulence factor as well as immunogenic antigen which can be used for immunization against *S*. Paratyphi A infections. The OSP lacks lipid A, since cleaved by ultracentrifugation, thus OSP are not visible by silver staining procedure. Our findings are in accordance with already published data relating to LPS and OSP of smooth colony forming bacteria in SDS-PAGE followed by silver staining (Konadu et al. 1999; Ali et al. 2012). The extracted LPS and derived OSP of *S*. Paratyphi A were checked for their purity level and found to contain 0.5% DNA and 1% protein contamination, thus conferring satisfactory purity level of these LPS and OSP of *S*. Paratyphi A.

The antigenicity of the purified *S*. Paratyphi A antigens, LPS and OSP was detected and these antigens were found to form evident precipitin lines in immuno-diffusion gels when tested against mice antisera raised to whole cell *S*. Paratyphi A. This showed that the antigenicity of the purified LPS and OSP was maintained. These results are in accordance with previous observations of antigenicity of LPS and OSP (Chu et al. 1991; Ali et al. 2014). Thus the purified OSP of the local isolate of *S*. Paratyphi A has the potential to be used as vaccine candidate alone or in conjugation with a carrier protein for immunization against paratyphoid fever in this region.

## Conclusion

The purified OSP antigen of *S*. Paratyphi A has the potential to be used as a vaccine candidate independently or may be conjugated with carrier protein to make low cost, specifically effective and immunogenic vaccine against paratyphoid fever.

## Abbreviations

LPS: lipopolysaccharides
OSP: O-Specific polysaccharides
SPA: *Salmonella* Paratyphi A
TSB: tryptic soy broth
PCR: polymerase chain reaction
DNA: deoxyribonucleic acid
SDS-PAGE: sodium dodecyl sulfate polyacrylamide gel electrophoresis
